# Can ‘local public food procurement’ extend to healthcare? – A policy content analysis

**DOI:** 10.3389/fnut.2026.1906366

**Published:** 2026-08-21

**Authors:** Kathy Faulkner, Jorja Collins, Heather Gilbertson, Judi Porter, Alexandra Chung

**Affiliations:** 1 Department of Nutrition, Dietetics and Food, Monash University, Melbourne, VIC, Australia; 2 The Royal Children’s Hospital, Melbourne, VIC, Australia; 3 Deakin University Institute for Physical Activity and Nutrition, Burwood, VIC, Australia

**Keywords:** catering, foodservice, healthcare, hospital, local procurement, policy analysis, public procurement, sustainability

## Abstract

**Introduction:**

Local public food procurement (LPFP) by hospital foodservice offers governments an opportunity to support local food systems and capitalise on multiple co-benefits. Policy is a potential lever to shift procurement practices. This study aims to identify and analyse food policy documents that promote LPFP, to explore how LPFP is represented, and the extent to which it extends to healthcare.

**Methods:**

Policies from the *World Health Organisation (WHO), Global Database on the Implementation of Food and Nutrition Action* (GIFNA) were screened for eligibility, descriptively characterised and qualitatively analysed.

**Results:**

Forty-two policies relating to LPFP were identified; almost 70% targeted schools, half were from Europe and a third were from Latin America and The Caribbean. Five overarching themes described how LPFP was represented in policies: LPFP benefits nutrition and local food culture, social economic development, and environmental sustainability; a multiplier effect where LPFP offers greater value than its initial investment; and variable policy commitment to LPFP. The representation of LPFP was influenced by regional, cultural and context specific factors. Ten policies (24%) included reference to hospitals, mostly described as one of many public institutions that could potentially support LPFP. Just one policy was specific for hospitals which represented LPFP for its environmental benefit.

**Conclusion:**

Overall, the extent to which local food procurement was recommended in healthcare was minimal. Findings suggest, that to establish LPFP in healthcare and realise its multiplier effect, it should to be prioritised under a strategic national strategy, to better enable a coordinated collaboration across the food supply.

## 1 Introduction

Public food procurement, has been recognised for its potential to support social development priorities, while simultaneously fostering transformative improvements across food systems (1). As significant and reliable purchasers of food for public institutions, governments hold considerable leverage to set limits around what food is purchased (minimally processed, nutritious, etc.), where it comes from (local etc.) how it is produced and transported (organic, animal welfare, air freight, etc.) and from whom it is purchased (smallholder farmers, local producers, minority groups, etc.), creating opportunity to deliver positive outcomes across a number of societal goals including population health and nutrition outcomes, environmental sustainability metrics, local employment, equity and social inclusion (2). Policy is typically the lever used to shift public food procurement norms. A number of international agencies and academic scholars endorse public food procurement as a strategic entry point for delivering food system co-benefits (1, 3, 4). Reeve et al. (5) identified and analysed global guidance reports to develop a framework aimed at supporting governments in formulating public food procurement policies. Examples of successful values informed public food procurement policies by national governments exist. In Brazil school meals must include a proportion of food sourced from family farmers and the inclusion healthy, safe, nutritionally adequate and culturally appropriate food is specified (6), and Denmark has achieved a transition of public kitchens to meet targets for organic food procurement (3, 7, 8). These examples are alike in that they are supported by overarching national strategies, with clearly defined procurement expectations, and alignment across policies and stakeholders.

While various different values can be emphasised by public food procurement, procurement of *local* food, is often promoted (3, 4). It is recommended for its ability to enhance food system resilience and economic development, while supporting environmental sustainability due to reduced transport emissions. Nutrition and health benefits from local food are also described including fresher food, less processed and from core food groups. Three main categories of ‘local’ food dominate; market-based definitions, e.g., purchase from family farmers; political boundaries, e.g., food produced within a county or region; and distance-based definitions, e.g., food produced within a specific distance of its point of its sale (9). Other academics such as Eriksen (10) offer a broader definition of ‘local food’, adding the category of values proximity, which includes for example, perspectives that promote local food for its ethical, social, environmental, and health attributes. This broader definition is useful because it simultaneously acknowledges that local food means “different things to different people in different contexts” (10) and that on some level, this contextual and idiosyncratic definition is part of its appeal. Indeed, a review of the purported benefits of local food systems by Enthoven and Van den Broek (11), found its impact on different social (including health), economic and environmental factors, to be highly case-specific and therefore difficult to compare. Despite refuting the idea that ‘local food systems’ are inherently good, they recommend, that if local food systems are to contribute toward a more sustainable food system transition, they need to be scaled, and their inclusion within public institutions could present such an opportunity for this.

Hospitals are influential public institutions. Traditionally, hospital food procurement prioritised ‘value for money’ but there is now a growing demand that food budgets be more strategically aligned with broader public and planetary health goals beyond minimum requirements (12, 13). In the UK, the National Health Service (NHS) identified healthier and more locally sourced food as a means of achieving a ‘Net Zero National Health Service’ (14). While in Australia, *The National Health and Climate Strategy* (15) recommends local food procurement by public healthcare as means of reducing climate emissions associated with food miles and an opportunity to support local economies. Yet, despite the demand, a scoping review of peer reviewed literature by Faulkner et al. (16) found just nine published examples of local food procurement in healthcare. The review revealed challenges with local food procurement for hospitals and farmers alike, including the logistics involved in working with multiple suppliers, difficulties for farmers in meeting hospitals’ year-around food demand and narrow specifications, and demanding administrative purchasing requirements. Despite the challenges, the review reported that hospital foodservice directors are supportive of purchasing local food.

Understanding policies that support local public food procurement (LPFP) may provide insights into how local food can be extended to healthcare. This could consider what policy drivers have been used, what level of commitment has been described, which settings (institutions) have been targeted, which government departments have been involved, and how has the issue been represented. The aim of this study is to identify and analyse food policy documents that promote LPFP and describe policy characteristics and representations of LPFP within policy documents, and determine the extent to which LPFP extends to public healthcare.

## 2 Materials and methods

A policy search and a qualitative content analysis was completed following three steps: (i) policy identification, involving a search and selection process, (ii) policy data extraction and characterization, and (iii) qualitative thematic content analysis to explore how LPFP is represented within and across identified policy documents.

### 2.1 Policy identification

#### 2.1.1 Search strategy

Policies were identified from the *World Health Organization (WHO) Global Database on the Implementation of Food and Nutrition Action (GIFNA)* (17) which contains information about nutrition policies and interventions from around the world, collected from a variety of sources or through users submitting their data. Policies were identified from two relevant sub-sets; *‘Public food procurement and service policies for healthy diets*’ (18) and ‘*Climate considerations in public food procurement*’ (19) and exported to an Excel spreadsheet (20) in Oct 2024 with descriptive information about each policy (title, country, year of publication) recorded. Complete policy documents were either retrieved from the database, or from a Google search, where the exact title was copied from GIFNA and put into the google search engine. The results from the google search were cross checked with the descriptive information available from GIFNA (date, title, country). If the Google search did not return a result after the first 10 hits, the policy was considered unavailable and was therefore excluded. The descriptive characteristics of the identified documents, including the results from the google searches (document found or not) are in Supplementary Table 1.

#### 2.1.2 Policy screening and selection

If required, policies were translated to English using Google Translate for documents. Any policy that could not be translated using this method was excluded, such as policies that were not digitised. All duplicates were removed. The title and executive summary of all retrieved policies were initially screened by one author for relevancy to public food procurement or local food, with any irrelevant documents excluded. A conservative approach was taken, and any borderline documents, or documents with inadequate information with which to make a decision, were included for further screening.

The next phase of the policy selection process was conducted independently in duplicate, involving a two-step process. Each policy was first screened for reference to ‘*public food procurement’* using a key word search with the following terms: *Procur (ement/ing), Purchas (e/ed/ing), Acqu (ire / asition), Supply, Source, Buy, Contract, Tender*). If a term was found and this section of the document related to *public food procurement,* the policy was then screened for any reference to *local food* using a key word search for the terms: *Local(ly), Season(al), Region(al), Origin, Tradition(al), Culture (al), Family farm(ing), Family agriculture, Indigenous, Native.* These broad search terms were used to ensure adequate search sensitivity, reducing the risk that relevant documents were excluded during the second screening process. If adequate reference to ‘local public food procurement’ was identified, the policy was included in the final data set. If no terms or minimal reference to *public food procurement* or *local food* was found in either step, the policy was excluded. Screening outcomes were compared among authors, and disagreements discussed until a final outcome was agreed upon. In cases where an agreement could not be reached, or where the document was considered borderline with reference to LPFP, a third author assisted with the final decision. Reliability was enhanced by having one author screen the entire set of documents. Tables 1, 2 describe the eligibility criteria. Supplementary Table 1 reports the screening and selection steps including the reason for document inclusion and exclusion.

**Table 1:** Eligibility criteria–public food procurement.

Include	Exclude
‘Procurement’ (or other listed term) found within the content of the document in the context of public food procurement (e.g., types of foods to procure, process or governance for procurement)	Does not describe procurement for a public institution (e.g., school, healthcare facility, prison etc.)
Just a brief mention of the term in the correct context	Procurement does not relate to food, i.e., might refer to equipment or supplier contacts
	Procurement is only referenced in terms of budget allocation
	Procurement is only described in terms of food safety

**Table 2:** Eligibility criteria–local food.

Include	Exclude
Local Food (or other listed term) found within the document and mentioned within the context of public procurement	Local food (or other listed term) not mentioned within the document
Public food procurement and local food are referred to within the document but may not be described in exactly the same place	Local food (or other listed term) clearly not associated with public procurement, e.g., nutrition issues that are common in the region, types of foods/dishes that are common to the region, procurement process to be adapted to local setting, local/regional governance of procurement,
	Minimal (superficial) reference to preferring local (or similar) food within the context of public food procurement

### 2.2 Data extraction and characterisation

Key characteristics were extracted from each policy and summarised in an Excel spreadsheet (20) including: global region (21), country economic classification based on World Bank income groups (22), year of publication, institutional setting, type of policy, involved government department(s) / agencies. Descriptive statistics were used to summarise and report the findings for each characteristic.

### 2.3 Qualitative thematic content analysis

A Constructivist approach was applied during analysis, recognising that policy is frequently developed in response to ‘problems’ that have been ‘constructed’ by the underlying social ideologies, causal beliefs and strategic objectives of different actors and alliances (23).

The framework method for qualitative content analysis described by Gale et al. (24) was used to determine how LPFP was represented across policies. Stage 1 involving interview transcription was not required and stage 2 involving familiarisation of policies occurred during data extraction and characterisation. Stage 3 and 4 involved developing a coding framework including testing and refining. The interrogating framework used by Jenkin et al. (25) to identify the ‘framing contests’ associated with obesity in New Zealand was modified to explore how LPFP is represented in policies. Three researchers independently used the coding framework to code five policies, purposely selected to demonstrate the breadth in policy characteristics. Differences in findings were discussed among the research team, and the coding framework was iteratively revised. One researcher repeated this process, supported by regular discussions and clarification regarding the coding process among the three researchers. The final coding framework is shown in Table 3. Stage 5 involved one researcher applying the coding framework to all remaining policies using an inductive approach. This involved reading and coding specific passages in policies that described values underpinning the directive to purchase local food. Stage 6 involved one researcher charting the data (coded passages) into the framework matrix (an Excel spreadsheet - see Supplementary Table 2) by organising codes (columns) and policies (rows). Illustrative quotes and examples of coded passages were included as evidence and to ensure that the underlying meaning was not lost. During the coding and charting process, contributing researchers regularly met to discuss progress. These meetings occurred more regularly during the initial stages of coding when clarification for any coding concerns was sought, discussed and recorded. Finally in stage 7, one researcher used the completed framework matrix to identify patterns and themes in the data. To do this, the coding framework was used to guide the categorisation of similar codes into themes under each interrogating question. Initial verification and support with interpretation were provided by another researcher who cross-checked themes during the development process. Themes continued to be generated iteratively by the lead researcher who met regularly with two other researchers in the team who reviewed and provided feedback until the final themes were agreed to be reflective of the data that had been charted. Finalised themes were compared across policies according to key characteristics (e.g., geographical region, policy type, country economic standing, government agencies).

**Table 3:** The coding framework.

Signature rhetorical devices	Prompts to identify and code data	Example codes
Position	How is local food described (key terms, definitions)?	Cultural, Traditional, Customs, Indigenous, Religious, Food sovereignty, Local specialities, Local, Short supply chain, Fewer intermediaries, Domestic Seasonal, Regional, Diversification of the region Family Farming
	What problems do ‘local public food procurement’ seek to solve / address?	National Food and Nutrition Insecurity Exposure to unhealthy, processed foods Poor nutritional status and educational outcomes of school students Lack of development, unemployment, poverty and food insecurity in regional areas
	Who benefits from ‘local public food procurement’?	School students, the broader school community Family Farmers, local producers and suppliers, Caterers Marginalised people: preferential employment of women, young people, ethnic minorities, Indigenous, etc. Broader community: economic growth, development, infrastructure Planetary health
Core values	What values or principles are evident in the way that ‘local public food procurement’ is represented as a solution?	Human Right to Healthy Food UN Rights of the Child Equality and/or Equity Intersectoral action Planetary Health
Solutions	How does the policy propose / include local public food procurement? What is the level of commitment to local public food procurement?	High Commitment includes: administrative, financial, multidisciplinary support, targets, mandating language Moderate Commitment includes: guidance for including local food in tenders and/or integrating within existing infrastructure, inspiring language Low Commitment includes policies lacking a strategy or justification for Local Public Food Procurement. Unclear / Unknown

#### 2.3.1. Sub-group analysis of policies that include or relate to healthcare

All policies that specifically mentioned healthcare, or included healthcare within the definition of an institutional setting, were analysed to identify the extent to which LPFP was related to healthcare, and any similarities in terms of characteristics and their representation of LPFP.

The whole authorship team reviewed the finalised results for the purpose of sense-checking.

### 2.4 Positionality statement

All researchers involved in this study are white, female and tertiary educated. Our professional backgrounds include the fields of nutrition and dietetics, food systems, food service dietetics, public food procurement, nutrition policy, management and tertiary education. Our team offers broad research experience including nutrition policy identification and thematic analysis. The authors acknowledge that their interpretation and portrayal of LPFP can influence this research. We are of the view that governments have the power to influence markets with their institutional spending. Further we believe that governments have the responsibility to move public food procurement for hospitals beyond minimum nutrient and food safety requirements to incorporate aspects of values-based procurement.

## 3 Results

### 3.1 Policy identification

**Figure 1:**
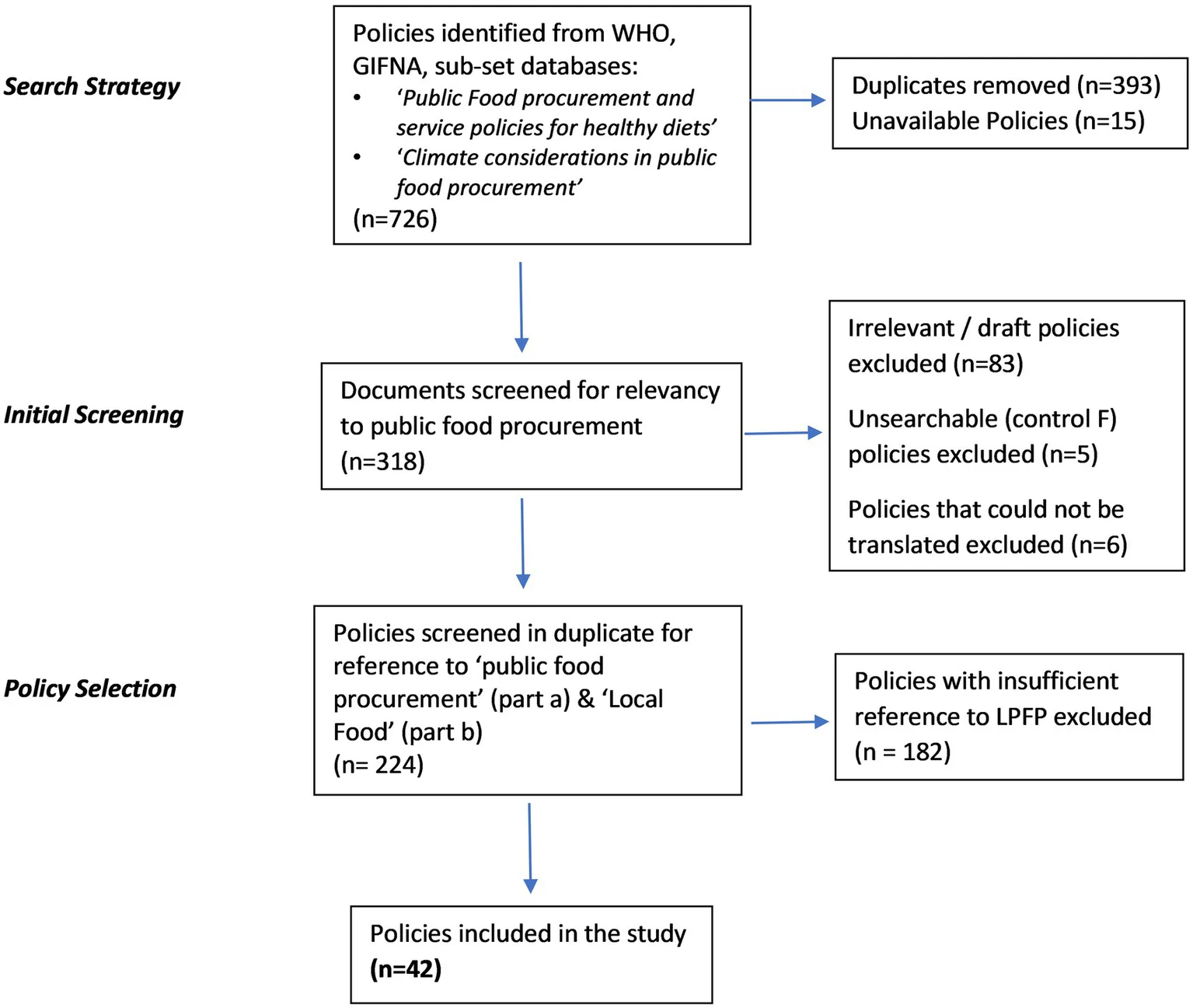
Summary of the search and screening process to identify relevant policies from WHO, Global Database on the Implementation of Food and Nutrition Action (15), describing local public food procurement.

Figure 1, describes the outcomes of the search strategy and screening process. From the 318 policies extracted from the GIFNA database, 42 (13%) were found to contain genuine reference to LPFP.

### 3.2 Extracted policy characteristics

Table 4 describes the key characteristics of the included policies. Policy documents were published between 2008 and 2024. The median publication date was 2017 with a large proportion of policies (43%) published in the 3 years between 2015 and 2017. Policies were derived from 31 countries across five global regions. Half of the policies were from Europe & North America and almost a third were from Latin America and the Caribbean. Three global regions were not represented including Australia & New Zealand, Northern Africa, Western Asia and Oceania. Some countries referenced LPFP within a number of different policies; they include Brazil (*n* = 5) (6, 26–29), Germany (*n* = 4) (30–33) Cabo Verde (*n* = 3) (34–36), Switzerland (*n* = 2) (37, 38), Slovenia (*n* = 2) (39, 40) and Slovakia (*n* = 2) (41, 42). Almost half of the extracted policies were from high-income countries (45%), and high and upper-middle-income countries represented more than 80% of all policies. Almost 70% of the policies were for educational settings, specifically schools and/or early learning settings. Two (5%) policies (42, 43) were specific to publicly funded institutions and just one policy was specific to hospital catering (31). Policies could be broadly categorised into four types; technical menu guidelines for institutional catering, legal documents (including laws, decrees and resolutions), procurement guidelines and nutrition related national policies. Technical menu guidelines were the most commonly identified policies and most (72%) legal documents also related to catering guidelines. Government ministries responsible for agriculture, education or health sponsored almost half of the policies, and another 17% were jointly sponsored by two or more ministries. For 24% of policies, the sponsoring government ministry was unclear. These policies tended to be legal documents published by National Governments (legislative or ministerial bodies, etc.) or they were authored by autonomous government agencies (e.g., Food Agencies). In addition to policies that were jointly sponsored by multiple ministries and Ministerial bodies, a number of policies (*n* = 6) involved strong inter-ministerial coordination and oversight (28, 29, 44–47).

**Table 4:** Characteristics of included policy documents.

Characteristic	Detail	Number (%)
Region	Europe and North America	21 (50%)
	Latin America and The Caribbean	13 (31%)
	Eastern and South East Asia	2 (5%)
	Central and Southern Asia	1 (2%)
	Sub-Sahara Africa	5 (12%)
	Australia and New Zealand	0
	Northern Africa	0
	Western Asia and Oceania	0
Economic classification (GNI/Capita)	Low-income ($1,135 or less)	1 (2%)
	Lower-middle-income ($1,136 – 4,495)	6 (14%)
	Upper-middle-income ($4,496 – 13,935)	16 (38%)
	High-income (more than $13,935)	19 (45%)
Date of publication	2008–2012	5 (12%)
	2013–2016	15 (36%)
	2017–2020	11 (26%)
	2021–2024	11 (26%)
Policy setting	Schools (nursery, primary, secondary)	29 (69%)
	Hospitals, aged care	2 (5%)
	Public institutions	2 (5%)
	Workplaces	2 (5%)
	Multi-setting – whole of community	7 (17%)
Leading government ministries	Ministry responsible for education	7 (17%)
	Ministry responsible for health	7 (17%)
	Ministry responsible for agriculture (Food)	5 (12%)
	Multiple ministries (Agriculture, Health, Education)	7 (17%)
	Other ministries: - Environment protection and regional development (1) - Family, youth and sport (1) - Home affairs (2) - Social and agrarian development (2)	6 (14%)
	Unclear: - Government (1) - National legislative bodies (4) - Ministerial bodies (2) - Autonomous government agency (3)	10 (24%)
Type of policy	Legal documents: - Relating to school catering (13) - Relating to work environment (1) - Public procurement guidelines (1) - National food and nutrition strategies (3)	18 (42%)
	National food security strategy	2 (5%)
	National obesity strategy	2 (5%)
	Public procurement guidelines	1 (2%)
	School nutrition policy	1 (2%)
	Technical catering/menu guidelines	19 (45%)

### 3.3 Themes

**Figure 2:**
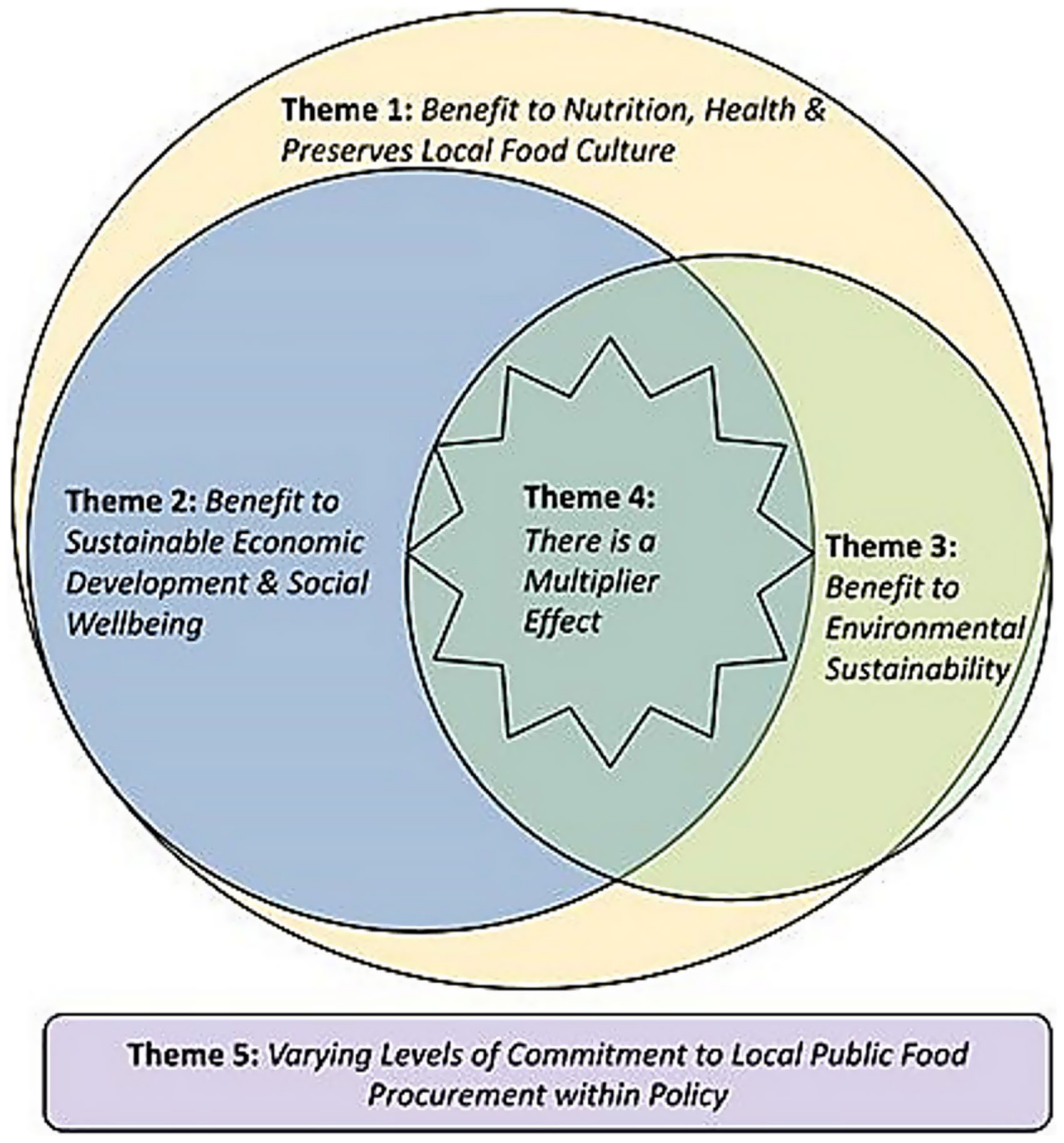
Themes arising from the data, illustrating how local public food procurement is represented within policies.

Five overarching themes (and 12 sub-themes) arose from the data, depicted in Figure 2. Four themes describe the benefit of LPFP; it promotes nutrition and health and preserves local food culture (theme 1); it supports sustainable economic development and social wellbeing (theme 2); it benefits environmental sustainability (theme 3); and theme 4 is the overlap between benefits and demonstrates the multiplier effect. Theme 5 describes the extent to which LPFP was committed to within policies.

A detailed description of the themes and sub-themes is presented below.

#### 3.3.1 Theme 1: local public food procurement promotes nutrition and health and preserves local food culture

##### 3.3.1.1 Sub-theme - local public food procurement promotes nutritional value, food safety, traceability, quality, taste, and seasonal diversity

Across almost all policies, LPFP was viewed as an opportunity to improve the nutritional value, quality and diversity of the food supply and meal service in public institutions. Local, seasonal, fresh fruits and vegetables were associated with superior quality, taste, safety, traceability and optimised nutritional density; having been grown with fewer pesticides and/or requiring less transport and storage than food purchased from further afield.

“*The advantage of domestic production is shorter transport and storage of fruits and vegetables, as well as the optimal ripening time, and all of this can contribute to higher nutritional value, as well as better quality and taste food*”. (48), p. 86

##### 3.3.1.2 Sub-theme: local public food procurement preserves traditional knowledge, cultural identity and seasonal rhythm

In this sub-theme, LPFP was represented as being able to help preserve traditional foods and recipes, which were considered healthier alternatives to the highly processed and/or imported foods commonly relied upon in institutional catering. This idea was particularly observed in policies from Latin America and the Caribbean. Local food was represented as promoting cultural identity and valuing food sovereignty and LPFP was used to encourage institutional catering services and recipients to re-discover traditional foods and recipes. Traditional foods were described as healthy and varied, responding to climate and season, benefitting the whole food system and aligned with concepts of sustainability, agricultural diversity and nutrient dense crops.

“*...rescuing traditional knowledge and know-how with local foods of high nutritional value, developing a food culture with a vision of nutritional food security with sovereignty*”. (49), p. 24

##### 3.3.1.3 Sub-theme: local public food procurement in school catering benefits student welfare, the wider school environment and the community more broadly

School meal programs (and the underpinning LPFP) were valued for their ability to improve student health, well-being and academic performance. The school meal program was often identified as a health promotion tool, capable of modelling healthy eating habits, local food culture, seasonal variety, food sovereignty and ecologically sustainable diets. Occasionally, the school meal program was described as a pedagogical tool, with potential to integrate with the school curriculum, establish connections with the local agricultural sector and encourage food literacy in students.

“*School catering should be considered an important and continuous moment of education and health promotion, aimed at children which also involves teachers and parents*” (50), p. 6

In low and lower-middle income countries especially, the school meal program was often identified as an important component of a country’s nutritional welfare system, valued for its ability to protect student health, prevent malnutrition, reduce school absenteeism and contribute to educational success.

#### 3.3.2 Theme 2: local public food procurement supports sustainable economic development and social wellbeing

##### 3.3.2.1 Sub-theme: local public food procurement supports the local food industry and creates employment opportunities

In low- and lower-middle-income countries, LPFP was an important enabler of the local food industry (agriculture, fisheries and/or processing). In some instances, the LPFP strategy targeted a specific industry, supplier or producer. Always, the goals were for sustainable development of the local food industry, creating employment, income and supporting agricultural diversification and productivity.

“*The development of the school nutrition system in the Republic of Tajikistan will allow: - creation of a guaranteed market for agricultural products, including products of local producers, increasing productivity and income levels in agriculture*”. (46), p. 11–12

As well as offering specific benefit to the local food industry, LPFP also offered other employment opportunities associated with the institutional meal program including of nutritionists, restaurateurs and local caterers or foodservice operators.

##### 3.3.2.2 Sub-theme: local public food procurement supports minority and vulnerable organisations involved in local food industry

Some policies, especially those from Latin America and the Caribbean encouraged preferential purchases from small and medium enterprises, family farmers and other less advantaged producer organisations such as those from ethnic and other minority backgrounds (6, 26, 28, 29, 35, 45, 49, 51, 52). These organisations were noted to benefit from a guaranteed, consistent and fair market, allowing for low-risk investment in their enterprises, encouraging innovation, modernisation, diversification, expansion and ultimately increased productivity and income.

“*…support for sustainable development, with incentives for the acquisition of diversified food products, produced locally and preferably by family farming and rural family entrepreneurs, prioritising indigenous communities and remnants of quilombos*”. (6) (Art. 2, V)

##### 3.3.2.3 Sub theme: local public food procurement supports regional and rural development

In this sub-theme, LPFP was represented to help address the problem of unemployment and poverty in rural areas. These policies sought to prevent regional decline by attracting and retaining youth, actively supporting the local food industry and affiliated opportunities including education. Some policies also identified concurrent improvements to regional infrastructure which was noted to be essential for improving efficiencies in domestic food production and supply, and offered far reaching regional community benefits.

#### 3.3.3 Theme 3: local public food procurement offers benefits to environmental sustainability

Specific reference to promoting LPFP on the grounds of environmental benefit was only observed in policies from high-income countries in Europe.

##### 3.3.3.1 Sub-theme: local public food procurement leads to fewer carbon emissions due to reduced transport and storage requirements

Local food was associated with reduced environmental impact due to reduced greenhouse gas emissions from transport (food miles) and storage.

##### 3.3.3.2 Sub-theme: local and seasonal foods are distinguished from ‘local alone’ for their reduced environmental impact

In some policies, the terms ‘local *and* seasonal’ together were used to describe food with a reduced environmental impact. Locally grown foods reduced the risk of air freight and long transport routes, while seasonal produce was less likely to be grown in hot houses; both reducing the risk of higher carbon emission. A few policies sounded a note of caution, noting that ‘local’, ‘regional’ and ‘seasonal’ are not ‘protected’ terms and their meaning can be misconstrued.

“*Seasonality represents the natural cycle of fruits and vegetables in their country of production. A “seasonal” product is therefore not produced in an artificially heated greenhouse. But be careful, “seasonality” does not necessarily rhyme with “regionality”. For example, Swiss tomatoes can easily be found in a heated greenhouse in winter: these are local but out of season and therefore not sustainable* (37), p. 49

##### 3.3.3.3 Sub-theme: local public food procurement represented as ethically superior (ecological, animal and social welfare)

Some policies identified local food as having been produced by agricultural or production methods that were ethically and ecologically superior. In these policies, valuing ethically produced food sought to increase demand for harm-reducing production methods, healthy ‘eco-smart’ diets, and local food purchase generally.

#### 3.3.4 Theme 4: the multiplier effect

Across most policies, LPFP was represented for its potential to offer multiple benefits to many beneficiaries, achieving a greater reach and value than its initial investment.

This multiplier effect was particularly evident in policies relating to school meal programs, where an investment in LPFP was seen to bring about concurrent improvements to students’ immediate nutritional and academic outcomes, their dietary habits and food literacy, while at the same time offering national benefit by supporting the local food industry, improving the domestic food supply and contributing to sustainable economic development.

“*…the development of further steps to provide students with healthy nutrition, develop educational potential, ensure food security, improve well-being of the population, and ensure the socio-economic development of the country*”. (46), p. 2

#### 3.3.5 Theme 5: the degree of administrative support shown for local public food procurement differs across countries and indicates varying levels of commitment

##### 3.3.5.1 Sub-theme: legal documents and National Strategies indicate high commitment to LPFP

‘Legal documents’ including laws, decrees and resolutions and National Food Security Strategies and Action Plans were the types of policies articulating higher commitment to LPFP. These policies also tended to align LPFP with supporting national food security, child nutrition welfare, the local food industry and improving the domestic food supply. This level of commitment to LPFP was absent in policies from European countries. Commitment to LPFP appeared as mandating language, specified targets for spending on LPFP, supportive coordinating committees with multidisciplinary representation, reduced administrative burden and/or where financial support is assigned.

##### 3.3.5.2 Sub-theme: administrative guidance within existing processes indicates moderate commitment to local public food procurement

Moderate commitment to LPFP existed in policies that offered administrative guidance for the integration of local food into public tenders and/or direction to exploit existing infrastructure and promote LPFP. This level of commitment existed across a range of different policies. In high income countries, policies used language that sought to inspire and enable rather than enforce. For example, these policies encouraged institutions to choose local and seasonal foods during peak seasons when supply is higher and costs are lower and they offered administrative guidance for the integration of local food in public tenders. In lower-middle income countries, moderate commitment to LPFP was represented as capitalising on existing local markets and promoting a more decentralised approach to procurement, a pilot/trial or period of transition, or specific future actions and dates.

“*With reference to short supply chain foods, it is useful for the Regions and Public Administrations to draw up a document listing some principles that help public administrations to define tender specifications capable of respecting the rules of free circulation of goods within the community, while simultaneously protecting freshness, zero-mile/short supply chain, local products (not necessarily yet classified as typical or traditional)*” (50), p. 19

##### 3.3.5.3 Sub-theme: policies lacking a strategy or justification for local public food procurement indicate minimal (low) commitment

Some policies included LPFP but did not describe any clear strategy for achieving it, or explanation for its recommendation. Overall, these policies offered minimal reference to local public food procurement. Other policies were extremely aspirational but offered no tangible support for LPFP. Some policies were unable to offer higher commitment due to minimal government oversight (i.e., the meal service is privately outsourced or inconsistently governed).

### 3.4 Policies that extend to healthcare

Ten policies (24%) from nine countries were found to include or relate to healthcare. Table 5, describes their key characteristics. Policies were from high- or upper-middle income countries (*n* = 6 and *n* = 4 respectively). Five policies mentioned hospitals specifically including policies from Brazil (28, 29) Carbo Verde (35), Slovenia (40) and Germany (31). Notably, these countries have all published other policies referencing LPFP included within this identified sub-set of policies. Germany has published a technical menu guideline specific to hospital catering, while the other policies encompass broader national nutrition priorities including food security (*n* = 3) (29, 35, 53), healthy eating guidelines (*n* = 2) (38, 54) and obesity prevention (*n* = 2) (28, 52). Five policies recommended LPFP for *institutional* catering (42, 43, 52–54). Two policies demonstrated strong commitment to LPFP; Brazil’s ‘National Plan for Nutrition and Food Security’ (29), and The Bahama’s ‘National Food and Nutrition Security Policy and Agenda for Action’ (53). These policies described inter-ministerial actions, mandated targets, supportive administration and infrastructure, and designated funding. Yet neither of these policies reflected a strong commitment to healthcare. Only, Germany’s ‘Quality standard for catering in clinics’ (31), was specific to healthcare. It included inspiring language and recommendations to include local food on hospital menus, quality improvement checklists and links to supportive resources, but offered minimal tangible support for the inclusion of local food on hospital menus. It is included as part of Germany’s IN FORM initiative, and administered by the Federal Ministry of Food and Agriculture (BMEL).

**Table 5:** Characteristics of policies that relate to healthcare including how they promote local public food procurement.

Country, year	Leading Gov ministry	Policy type/setting	Representation of local public food procurement in healthcare/institutions
Bahamas, 2017	The ministries responsible for health and agriculture	National food security strategy and action plan Multi-setting including public institutions	Local public food procurement is represented as a means to improve food and nutrition security within existing food systems. Supported by: An intersectoral coordinating commission Integration across existing programs and institutions Named actions and targets with associated funding
Brazil, 2014	CAISIN^1^ – Interministerial chamber of food and nutrition security chamber (Led by ministry responsible for social development)	National obesity prevention plan Multi-setting – hospitals specifically named	Local public food procurement is represented as supporting traditional, healthy eating habits and as an opportunity to support family farmers. Describes examples of support local public food procurement by different levels of government including: Inter-sectorial opportunities Training/CPD Improvements to existing local food collection centres (ceasas) Laws promoting institutional food procurement from family farmers
Brazil, 2017	CAISIN^1^ – interministerial chamber of food and nutrition security chamber (Led by ministry responsible for social development)	National food security strategy and action plan Multi-setting – hospitals specifically named	Local public food procurement is represented as an opportunity to encourage family farming and improve the food supply. Supported by: Regulation – 30% of the institutional food budget to be spent on food from family farms Administrative support Intersectoral government action Funding for training and supportive infrastructure.
Cabo Verde, 2015	National assembly CNSAN^2^ – Advisory body on food and nutrition security	Legal document – national food security strategy Multi-setting – hospitals specifically named	Local public food procurement is represented as an opportunity to promote sustainable, productive and diversified agriculture. Supported by strategic objectives including: To promote consumption of local foods in state institutions To adapt public procurement legislation to local production conditions.
Dominican Republic, 2017	The ministry of public health	National obesity plan Multi-setting including public institutions	Local public food procurement is represented as an opportunity to support family farmers to upscale production of fresh, safe, nutritious food especially fruit and vegetables. Supported by: One Action (5.1.2) assigned to multiple government entities
Germany, 2022	INFORM - Germany’s initiative for healthy nutrition and more exercise (Overseen by the federal office for agriculture and food)	Technical menu guideline Addressing hospitals and rehabilitation clinics	Local food is represented as safer, higher quality and ecologically beneficial. Inspiring those responsible for catering to include local food on hospital menus through: Recommendations and quality improvement check-lists Links to supportive resources
Latvia, 2017	Cabinet of ministers	Legal document - procurement guideline All public institutions	Local public food procurement is represented for its ecological benefit. Supported within a technical guideline for writing a ‘Green Public Procurement’ tender including: Definitions and exceptions Compliance requirements Evaluation criteria
Portugal, 2017	Interministerial council (The departments of: Education, Health, Economy, Agriculture, Forestry and Rural Development and Sea)	Legal document -national healthy eating strategy Multi-setting including public institutions	Local food is associated with a traditional Mediterranean (healthy) diet. Local public food procurement is slightly represented as promoting innovation & entrepreneurship. Supported by recommendations to include local food: at public events for public catering
Slovakia, 2016	Ministry responsible for agriculture	Procurement guideline, All public institutions	Local food is represented as high quality, fresh and safe (microbiological and chemical contamination) and traceable. Local public food procurement is recommended but there is no actual commitment.
Slovenia, 2015	Ministry responsible for health	Legal document - national nutrition and physical activity program for health Multi-setting - hospitals specifically named	Local public food procurement is represented as promoting a safe, nutritious, and ecologically sustainable food supply. Recommended within: Priorities and strategic objectives A grocery ordering system for public institutions Actions assigned to specific government ministries and integrated with other policies

^1^CAISIN–Câmara Interministerial de Segurança Alimentar e Nutricional. ^2^CNSAN - Conselho Nacional de Segurança Alimentar e Nutricional.

## 4 Discussion

This study sought to explore local public food procurement (LPFP) and its presence and representation in food policy documents. Recommendations to include local public food procurement were identified in 42 policies across 31 countries from within the WHO GIFNA database, highlighting the popularity of this initiative across the world. Three benefits of LPFP were identified in policies, and the way that LPFP was represented tended to align with the priorities of the region. All policies (except one) represented LPFP for its ability to improve nutritional outcomes, which aligns with expectations of food policy documents. In Latin America and the Caribbean, LPFP was most often represented for its ability to support small-holder farmers, while in Europe it was represented for its ability to improve environmental sustainability metrics.

The majority of policies relating to LPFP identified in this study were from the European Union (EU). While EU public procurement rules prohibit explicit preference for local food, policy frameworks developed and promoted by the EU enable its indirect preference by aligning concepts of environmental sustainability, freshness, and food production methods that favour local producers without violating anti-discrimination laws (55, 56). This policy analysis identified similar themes in policies from European countries, which again reflects the contextual and regional nature guiding policy development. In Germany, the only country to have developed a policy specifically targeting healthcare, the national strategy known as IN FORM supports improvements to institutional catering, among other health promotion initiatives. While presented as recommendations and guidelines, they are supported by cross-sector coordination and policy coherence, and are jointly led by the ministry responsible for agriculture and food and the ministry responsible for health (57). Denmark has also been widely recognised for its transformative public food procurement policies. Denmark’s lack of a universal school meal program means it has not been able to rely on schools as the entry point for change and instead has had to focus on other institutional settings. Denmark has had greatest success in transforming smaller institutions such as childcare centres and workplace cafeterias, highlighting the fact that change in institutions other than schools is possible, although:

“*Whereas the implementation of organic foods has developed quite smoothly in smaller institutions such as kindergartens and nurseries, the introduction of organic foods into large-scale foodservice such as that found in hospitals and larger residential homes for the elderly, has proven to be quite difficult. One of the reasons for this is the highly complex planning, procurement and processing procedures pursued by such facilities*”. (57) (Introduction, paragraph 2)

Denmark’s mechanism for transformation includes establishing purchasing targets from centralised procurement systems, municipal coordination, kitchen-level transformation, and staff training (8). While Denmark’s focus is to increase the proportion of organic food purchase, the premise and mechanism for public food procurement transformation could be applied to other values such as local food.

Varying levels of commitment were observed in policies, which may influence whether LPFP is realised or not. The findings arising from this policy analysis suggest that regional coalitions, overarching national strategies, technical support from inter-governmental organisations such as the UN, as well as regional leadership by exemplar countries are supportive levers for context specific policy development and implementation. Brazil provides an example of strong policy commitment where public catering exists within a legally mandated national food security system; a highly structured framework that offers a platform for collaboration and action amongst different sectors and levels of government and civil society organisations (58). Within this framework, healthcare procurement is included under the ‘Food Acquisition Program’ (Programa de Aquisição de Alimentos—PAA, 2003)’ which aims to simplify the bureaucratic procurement process associated with institutional procurement allowing for the direct acquisition of food from family farmers by hospitals, becoming mandatory in 2016 (59). Yet the PAA has been vulnerable to changing political environments and was eventually disbanded in 2023. Authors Barbosa Jr. and Coca (60) discuss the political nature of public procurement policies in Brazil, noting that whereas the PAA has succumbed to government attack, the Brazilian School Meal program (PANE) has shown more resilience, which they argue is due to its framing, ‘support for school children’, its subsequent broad social appeal and the fact that it is embedded within the ‘untouchable’ education budget. They conclude that for public procurement policies to succeed beyond political administration cycles, they need to be anchored within secure government budgets and offer broad social appeal.

Schools were the leading setting for local public food procurement across policies. School feeding programs represent broad scope to provide social welfare and can have an important role in combating child hunger (3, 61). They are promoted by the United Nations World Food Program (WFP), and the Food and Agricultural Organisation (FAO) and are especially prevalent in Latin America and the Caribbean and Sub-Sahara Africa where they are further supported by regional coalitions and strategies (62, 63). School meal programs have been evolving since the early 2000’s from a food-aid model to a strategic movement improving food systems by linking nutrition outcomes with local agricultural development (64). Brazil’s National School Feeding Programme (Programa Nacional de Alimentação Escolar – PNAE, 2009) exemplifies this. As of 2025 at least 45% of food for school meal programs must be purchased from smallholder farmers (6). The benefit of local food purchase by schools is represented for its multiplier effect, as a means of supporting local agriculture and in particular small holder farming, while at the same time improving the nutritional value of the food supply in schools, and improving student nutrition and educational opportunities and outcomes (61, 62). While schools are an important institutional setting for creating food systems change (63), for countries that do not have school meal programs other institutions must be considered.

The opportunity to harness public hospital food procurement for food system change is significant; a recent report from Australia estimates that nationally publicly funded hospitals spend AUD 345 million per year on food procurement, second only to that of residential aged care (65). But despite this opportunity, this policy analysis found that the extent to which policies recommended LPFP for healthcare was minimal, mentioned as one of many institutional settings within national nutrition policies and public procurement guidelines. Only one policy was identified in this analysis as being specific to healthcare, a catering guideline for hospitals, funded by the Ministry responsible for Agriculture and Food, as part of Germany’s IN FORM initiative (56). In this policy local food was strongly aligned with improvements to environmental sustainability metrics (31). Indeed the opportunity to represent local food as a mechanism for achieving the goal of net zero emissions for healthcare is promising and there are a number of relevant climate policies from developed countries promoting this action (14, 15). There is also strong advocacy from not-for-profit organisations and networks, providing technical support for ‘on the ground’ transitions (66). Furthermore, a scoping review by Alberdi and Begiristain-Zubillaga (12) positions local food as an important strategy to achieving sustainable food procurement for healthcare. The review acknowledges the many challenges facing hospital food procurement conversion, not least its current reliance on procurement and distribution systems which have evolved to be efficient on the basis of price, convenience and complex bureaucratic administration. Loss of kitchen infrastructure and staff skill are an unfortunate outcome. Additional food safety requirements, scale, narrow quality specifications and inflexible menu design further challenge local food suppliers in their ability to meet hospital demand (16, 67). While researchers offer many recommendations to overcome challenges, Mikkelson (67) notes of Denmark’s organic kitchen conversion, that political framing was an important initiator of the conversion process and that “without political support conversion would not have been possible.” Indeed, the successful public food procurement programs in Brazil and Denmark indicate that change is feasible with shared political will, effective use of policy and concerted coordinated effort. The findings from this analysis suggests that a high level of policy commitment to LPFP exists when LPFP is included as part of a national framework, with long-term commitment and resourcing across multiple, shared government priorities, which is expected to better enable downstream policy cohesion and collaboration among stakeholders. Alberdi and Begiristain-Zubillaga (10) assert that a systems approach is required for adopting sustainable public food procurement strategies, important for preventing a siloed mentality and for obtaining a multiplier effect across the food system. The findings from this policy analysis appear to corroborate this assertion, all-be-it in relation to school meal programs, where an investment in LPFP was represented to offer multiple concurrent improvements to students’ immediate nutritional and academic outcomes, their food literacy, the food supply and to sustainable economic development. Likewise, within the healthcare setting, LPFP could be promoted for its purported multiple co-benefits, including for example, improvements to the quality of hospital food (promoting patient nutritional intake and recovery), support for local industries (promoting economic development and resilience in local food supply) and a reduction in carbon emissions from transport (aligning with climate goals). Finally, LPFP could be represented as supporting the moral imperative for hospitals, as trusted health-promoting organisations, to ‘lead-by-example’ and model healthy and sustainable food environments (12).

### 4.1 Strengths and limitations

This policy analysis offers an in-depth thematic interrogation of global nutrition policy documents involving a team of nutrition and dietetics researchers with a wide breadth of expertise and experience across food systems, public food procurement and nutrition policy. There are numerous strengths to the policy search, including the absence of both date and language restrictions. However, no policies were identified from three geographic regions (Australia, New Zealand and Oceania, Northern Africa, and Western Asia). This may reflect limitations of the policy database used, rather than a genuine lack of policies from these regions relating to LPFP. Policies within GIFNA are identified by searches conducted by the WHO or partner organisations or through direct submissions from countries or representatives, which may bias particular regions. A language and regional bias may additionally have been introduced due to the exclusion of documents that could not be identified from google searches, machine translation, or internal electronic searches. Another limitation of the GIFNA database is its inherent focus on nutrition, with policies referencing LPFP outside of nutrition-focussed domains not captured. The variability of the retrieved policies which included technical menu guidelines, broad national nutrition policies, public purchasing guidelines and technical legal documents, as well as the limitations of the electronic translations made the thematic analysis challenging at times. Also, inherent in any thematic analysis is the chance for researcher bias which was reflexively managed through regular discussions by the team of experienced researchers and their broad nutrition expertise. Finally, policy analysis, as a standalone research method, is unable to elicit if LPFP is actually occurring as stated. We tried to address this limitation to some extent by extracting and reporting on the level of commitment each policy had to LPFP.

The representation of LPFP within policy documents highlights significant regional and cultural influences, and the importance of context specific policy development. Representation of LPFP for its multiple co-benefits may help to progress its agenda within institutions, offering alignment with any number of social (including health), environmental and economic incentives. Furthermore, findings suggest that policy is most likely to succeed when it is included as part of a national framework, with long-term commitment and resourcing across multiple, shared government priorities, which is expected to better enable downstream policy cohesion and stakeholder collaboration. In order to establish LPFP and realise its multiplier effect, the findings from this analysis suggest it should be prioritised under a unifying national strategy to better enable coordinated collaboration across the food supply.

## Data Availability

The original contributions presented in the study are included in the article/Supplementary material, further inquiries can be directed to the corresponding author/s.
